# 1663. Risk-benefit assessment of cefazolin use for surgical site infection prevention among patients allergic to penicillins and cephalosporins

**DOI:** 10.1093/ofid/ofac492.129

**Published:** 2022-12-15

**Authors:** Meghan N Jeffres, Melissa Porter, Madison Ricco, Miranda Norvell, Craig Hogan, Eric Bassler, Ryan Koonce

**Affiliations:** University of Colorado Anschutz Medical Campus, Aurora, Colorado; University of Colorado Skaggs School of Pharmacy, Aurora, Colorado; University of Colorado Skaggs School of Pharmacy, Aurora, Colorado; UCHealth University of Colorado Hospital, Aurora, Colorado; University of Colorado School of Medicine, Auroa, Colorado; University of Colorado School of Medicine, Auroa, Colorado; University of Colorado School of Medicine, Auroa, Colorado

## Abstract

**Background:**

Prosthetic joint infection (PJI) is a devastating complication following knee and hip arthroplasty. Perioperative antibiotics are routinely used in an effort to decrease the risk of surgical site infections (SSI), including superficial skin infections and deep PJI. The American Academy of Orthopedic Surgeons recommend cefazolin as standard of care for SSI prophylaxis. However, non-beta-lactams are often used in patients labeled as allergic to penicillins or cephalosporins due to concern of increased hypersensitivity reactions (HSR).

**Methods:**

This study examined the frequency of SSI and HSR after receipt of perioperative cefazolin compared to clindamycin and/or vancomycin (C/V) within adult patients labeled as allergic to penicillins or cephalosporins. Patients were included if they underwent primary total hip or knee replacement surgery between January 2020 and July 2021 within the University of Colorado Health system. The outcome of SSI was assessed up to 90 days after surgery. The outcome of HSR was assessed up to 60 minutes after the conclusion of surgery.

**Results:**

The cohort included 1,128 patients (cefazolin, n=809; C/V, n=319). Baseline characteristics were similar between groups except the proportion of patients with a history of immediate allergy symptoms was greater in the C/V group (59.6%) compared to the cefazolin group (35.8%). The frequency of SSI was lower in the cefazolin group compared to the C/V group (0.9% vs. 3.8%, p < .01). The cefazolin group included 7 SSI, consisting of 6 superficial SSI and 1 deep PJI. The C/V group included 12 SSI, consisting of 6 superficial SSI and 6 deep PJI. HSR were not significantly different between patients receiving cefazolin vs. C/V (0.2% vs. 1.3%, p=.06). HSR in the cefazolin group consisted of skin involvement (n=1) and self-reported allergy in post-operative window (n=1). Both patients had a non-descript history of penicillin allergy. HSR in the C/V group consisted of skin involvement (n=2) and hemodynamic instability (n=2). Three of the four patients had a history of immediate cephalosporin allergy.

**Conclusion:**

Among penicillin or cephalosporin allergic patients, the use of cefazolin was associated with a lower frequency of PJI without an increase in HSR during surgery.

**Disclosures:**

**All Authors**: No reported disclosures.

